# Attitude in Radiographic Post-Operative Assessment of Dental Implants among Italian Dentists: A Cross-Sectional Survey

**DOI:** 10.3390/antibiotics9050234

**Published:** 2020-05-07

**Authors:** Bianca Di Murro, Piero Papi, Pier Carmine Passarelli, Antonio D’Addona, Giorgio Pompa

**Affiliations:** 1 Department of Oral and Maxillo-Facial Sciences, “Sapienza” University of Rome, Caserta 6, 00161 Rome, Italy; bianca.dimurro@uniroma1.it (B.D.M.); giorgio.pompa@uniroma1.it (G.P.); 2 Division of Oral Surgery and Implantology, Department of Head and Neck, Oral Surgery, and Implantology Unit, Institute of Clinical Dentistry, Fondazione Policlinico Universitario A. Gemelli IRCCS-Università Cattolica del Sacro Cuore, 00168 Rome, Italy; piercarminepassarelli@hotmail.it (P.C.P.); antonio.daddona@policlinicogemelli.it (A.D.)

**Keywords:** survey, dental implants, radiographic imaging, periapical X-rays, attitude, retrograde-peri-implantitis

## Abstract

There is a lack of evidence in the attitude and prescribing practice of implantologists in dental implant post-operative assessment; therefore, the aims of this cross-sectional study were to investigate these habits and the knowledge about radiographic aspect of retrograde peri-implantitis (RPI) among Italian implantologists. A questionnaire was sent via email to dentists randomly selected from the register of implantology and oral surgery societies. It included three questions: the preferred X-ray after implant placement, the timing of post-operative assessment and the knowledge of the RPI radiographic representation. A final sample of 434 implantologists was included in the study. The majority of them (84.3%) perform a periapical X-ray as control radiograph and picked the correct radiographic representation of RPI (74.3%), without statistically significant differences (*p* > 0.05) for sex, age, years of working practice and number of implants placed per year. Just 47.7% of dentists perform a control radiograph at prostheses delivery, to establish a proper baseline. A statistically significant difference (*p* < 0.05) was detectable only for the number of implants placed per year, with dentists placing > 80 implants selecting the correct choice. To the best of authors’ knowledge, this is the first study to report data on attitude of implantologists in radiographic imaging after implant placement.

## 1. Introduction

Implant prosthetic rehabilitation has become widespread in clinical practice over the last 20 years, with dental implants showing great success rates in the management of complete or partial edentulism [1,2,3]. Periapical and panoramic radiographs represent accessible, quickly and low-cost imaging technology available in almost every dental office: they are used in implant dentistry mainly for pre-surgical planning, to evaluate the height of bone available to place an implant without damaging critical anatomic landmarks, such as the maxillary sinus floor or the alveolar inferior nerve [4,5]. Furthermore, after implant placement, they allow for the reproducible and objective measurements of peri-implant bone levels, an important indicator of peri-implant health and a reliable tool for preventing implant failure [6,7,8]. To compare bone changes over time, radiographic film position must be standardized (Rinn, York, PA, USA) and lighting settings have to be similar to minimize results alterations [9]. Digital radiographs, such as phosphor-plate and RadioVisioGraphic (RVG) systems, allow for an easy standardization of contrast and, after the exposure, the images can be enlarged to facilitate the measurements [9]. The main limitation of periapical radiographs or orthopantomographs is the 2-dimensional images obtained, which clearly show only the interproximal area [5]. 

Today, 3-dimensional imaging techniques, such as cone beam computed tomography (CBCT) can improve the determination of bone height and thickness, the detection of anatomical structures, and represent the second-level imaging which is most used in dentistry [5,10]. The main difference between the classic computed tomography (CT) and CBCT is the acquisition mode of images, with a fan-shaped beam for CT capturing limited thickness slices and a cone-shaped beam for CBCT [11,12]. Moreover, the CBCT is quicker and can be accomplished at a lower radiation dose [13,14]. The CBCT is certainly more accurate for preoperative planning, compared to standard two-dimensional X-rays, however the higher radiation dose received by patients and the major cost constitute important drawbacks [15,16]. For these reasons, periapical radiographs should be considered as the “gold standard” for scientific evaluation [5], representing a widely accepted method to assess the evaluation of interproximal crestal bone changes of dental implants over time [17,18,19].

The important role of periapical radiographs to detect the condition and the quantity of bone around the implant surface is incontestable. A radiograph after surgery is important to find out the correct insertion and direction of the implants; in addition, according to the 2017 World Workshop on the Classification of Periodontal and Peri-Implant, diseases and conditions were adopted to establish the diagnosis of peri-implant disease [20]; a radiograph should be performed at the time of prosthetic loading to be used, as a baseline evaluation to determine alveolar bone levels over time. Peri-implant health is defined as the absence of clinical signs of inflammation, bleeding and/or suppuration on gentle probing, without radiographic bone loss visible on radiographs. Hence, radiographic imaging is important to classify implants as clinically healthy, since the corono-apical loss of supportive bone around an osseointegrated implant is a well-known indicator of peri-implantitis [20]. 

**Figure 1:**
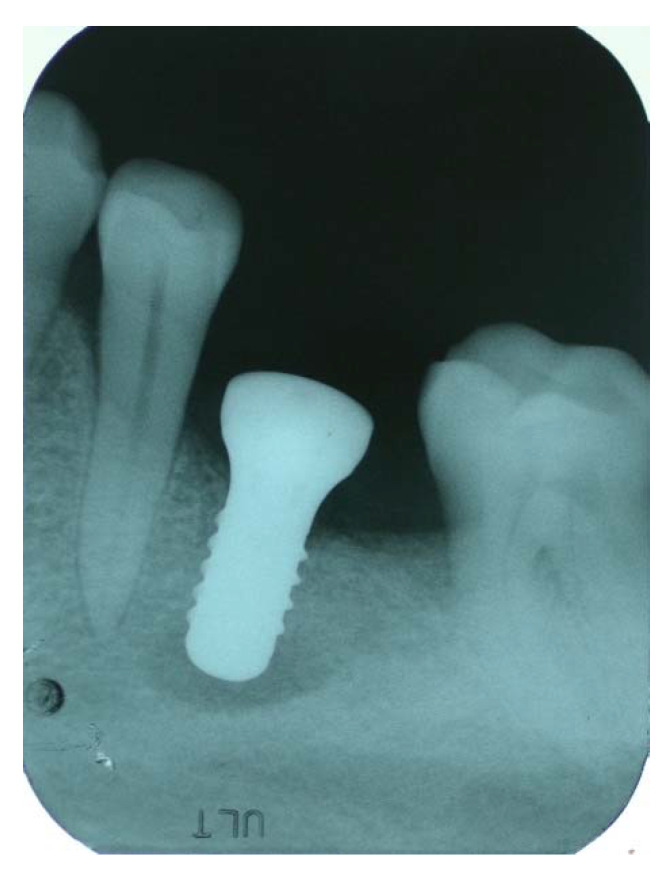
Radiographic aspect of retrograde peri-implantitis.

There is another pathological condition, named retrograde peri-implantitis (RPI) [21,22], which represents a relatively unknown disease that could lead to implant failure. This lesion appears as a radiolucent area, only around the apical part of the implant (Figure 1), with an apico-coronal pattern of bone resorption, and in most cases, the diagnosis could be performed only by imaging within the first 8 weeks after implant placement [23,24,25]. Therefore, the ability of the implantologist to recognize retrograde peri-implantitis as an apical radiolucent lesion around the apex of the implant is extremely important. RPI prevalence seems to be very low (0.26%), compared to marginal peri-implantitis; however, an increase up to 7.8% was reported when an implant’s neighbouring teeth exhibited an endodontic infection [24,25].

Just a few studies [26,27] have investigated the prescribing preference of dental practitioners in dental implant assessment, while, on the other hand, there is currently a lack of evidence in the attitude and prescribing practice of implantologists in dental implant post-operative assessment. Therefore, the aims of this cross-sectional study were to investigate the radiographic prescribing habits of implantologists in evaluating post-operatively dental implants placed and their knowledge about the radiographic aspect of retrograde peri-implantitis.

## 2. Results

### 2.1. Participants

Out of the 937 dentists contacted, a total of 516 subjects completed the survey (55.06%); eighty-two answered “no” to the question “Do you place dental implants on a regular basis?” and were, therefore, excluded. A final sample of 434 dentists was included in the study, they were either males (n = 386, 88.9%) or females (n = 48, 11.1%). The age range most represented was 45–64 years (50.2%), then 30–44 years (26.3%), < 30 years (18%) and > 65 years (5.5%). 

The relative majority of dentists enrolled had > 19 years of experience (44.7%) and placed 20–80 dental implants per year (49.1%), with all demographic characteristics described in Table 1.

### 2.2. Type and Frequency of X-ray

The first question was about the radiographic method performed after implant treatment: the vast majority of dentists (n = 366, 84.3%) take periapical X-ray, while 38 (8.8%) a panoramic and 30 (6.9%) a CBCT. No statistically significant inter-group differences (*p* < 0.05) were detected based on age, years of experience and number of implants per year (Table 2).

The question “when do you perform the control X-ray after implant placement?” could be answered by selecting multiple responses between the following ones: after surgery, after 1-3 weeks, at implant uncovering, at prostheses delivery. Interestingly, only 206 dentists (47.7%) selected “at prostheses delivery” among possible answers, with a statistically significant inter-group association (*p* < 0.05) with the category > 80 dental implants per year. There were no statistically significant inter-group differences (*p* > 0.05) among answers for sex, age and years of experience (Table 3).

As for the other answers, the vast majority of dentists (n = 362, 83.4%) included in their answers a control X-ray immediately after implant placement, while just 94 (21.6%) after 1-3 weeks from implant placement and a control X-ray at implant uncovering is made by 250 dentists (57.6%).

### 2.3. Retrograde Peri-implantitis

The last question was related to retrograde peri-implantitis: “how is the radiographic aspect of retrograde peri-implantitis?” The majority of dentists (n = 318, 74.3%) picked the correct answer “a radiolucent area at the apical aspect of the implant”, while 74 (17.3%) selected “a radio-opaque area at the apical aspect of the implant” and 36 (8.4%) “a radiolucent line at the lateral side of the implant”.

No statistically significant inter-group differences were found for age, years of experience and number of implants placed (*p* > 0.05) (Table 4).

## 3. Discussion

Over the years, radiographic imaging has become a fundamental tool in dentistry, in particular in implant dentistry, where it is used to assess bone height, bone resorption, the proximity of the maxillary sinus floor, and/or the neurovascular landmarks [4,5].

The aim of this cross-sectional survey was to investigate the attitude and the habits of a population of Italian dentists in radiographic imaging in implant dentistry and their knowledge about the radiographic aspect of retrograde peri-implantitis. 

Based on the results of our study, intraoral periapical X-ray is the most widespread radiography (84.3%) performed to evaluate the accuracy of implant placement. Nevertheless, a small number of dentists (8.8%) selected panoramic radiograph as their most commonly used control X-ray, even though it is characterized by a lower reliability due to its non-linear distortion and low spatial resolution [28].

On the other hand, another minority of clinicians (6.9%) choose CBCT, a radiographic method which should be dedicated to specific postoperative complications (such as neurovascular trauma or following more complex surgical procedures), and limited as much as possible to decrease the higher radiation dose received by patients [29].

The ALARA principle (as low as reasonably achievable) should be carefully taken into account when selecting the most appropriate radiographic technique for every situation, considering that only no exposure to ionizing radiation can be classified as completely free of risk [30].

According to Recommendation 8 of the position statement of the American Academy of Oral and Maxillofacial Radiology on criteria for the use of radiology in implantology [31], perapical X-ray should be used for the postoperative assessment of dental implants, while panoramic radiographs may be indicated for cases with multiple implants. According to recommendation 9 and 10, CBCT should not be used for periodic assessment of asymptomatic implants and postoperatively only, in case of altered sensation or implant mobility.

The vast majority of dentists (83.4%) perform an X-ray immediately after surgery to check the position and orientation of implants. An X-ray immediately after prostheses delivery helps clinicians to evaluate the long-term status and prognosis of the implant with a reliable baseline, allowing for reproducible and objective measurements of peri-implant bone levels [32]. For these reasons, the answer “at the time of prostheses delivery” was considered as the correct answer, but, interestingly, only less than half (47.7%) of dentists included in the survey selected this choice in their answers.

A statistically significant association (*p* < 0.05) was found for the variable number of implants per year, with the vast majority of dentists placing more than 80 implants/year selecting this answer.

No statistical differences were found for the other variables considered (sex, age and years of experience). Based on the last World Workshop (2017) on the Classification of Periodontal and Peri-Implant Diseases and Conditions, case definitions for day-to-day practice define peri-implantitis as presence of bleeding and/or suppuration on gentle probing, with increased probing depth compared to previous examinations and the presence of bone loss beyond crestal bone level changes resulting from initial bone remodeling [20]. Therefore, a baseline periapical X-ray is extremely important for an early detection and treatment of peri-implantitis, only in absence of a previous radiographic examination the criteria change and radiographic bone levels ≥ 3 mm apical of the most coronal portion of the intra-osseous part of the implant are considered consistent with a diagnosis of peri-implantitis [20].

Retrograde peri-implantitis is considered almost an unknown disease [25], therefore, we included a question about its radiographic representation. Based on the results of this survey, the vast majority of dentists correctly answered this question, however a consistent minority of 25.7% selected wrong answers, with no statistically significant association (*p* > 0.05) for variables evaluated (Table 4). Retrograde peri-implantitis could lead to early implant failure if the apical bone resorption extends to the coronal aspect of the implant. The most common symptoms include swelling, abscess, fistula and pain in the implant area. In the majority of cases, signs appear within the first weeks after implant placement [25], however, frequently patients are asymptomatic and retrograde peri-implantitis is only diagnosed after a routine radiographic examination. Therefore, the ability of the implantologist to recognize retrograde peri-implantitis as an apical radiolucent lesion around the apex of the implant is extremely important. Based on these findings, performing a periapical X-ray in the first 3 weeks after placement or at implant uncovering might help clinicians to intercept signs of retrograde peri-implantitis.

The main limitation of the study is represented by the difficulty to control the authenticity of the answers obtained: even if the questionnaire was administered via email and completely anonymous, we do not know if the answers selected matched the real behavior of dentists interviewed in clinical practice.

## 4. Materials and Methods

### 4.1. Study Design

To address the research purpose, the authors developed and implemented a cross sectional study, based on a self-designed survey, conducted at the Department of Oral and Maxillo-Facial Sciences, at “Sapienza” University of Rome in June 2019. The study was reported in accordance with the STROBE statement (www.strobe-statement.org). All questionnaires were anonymous and no personal data of participants was collected, therefore, ethical approval was not required based on the guidelines of our Institution Review Board (“Sapienza” University of Rome).

### 4.2. Study Population

**Figure 2:**
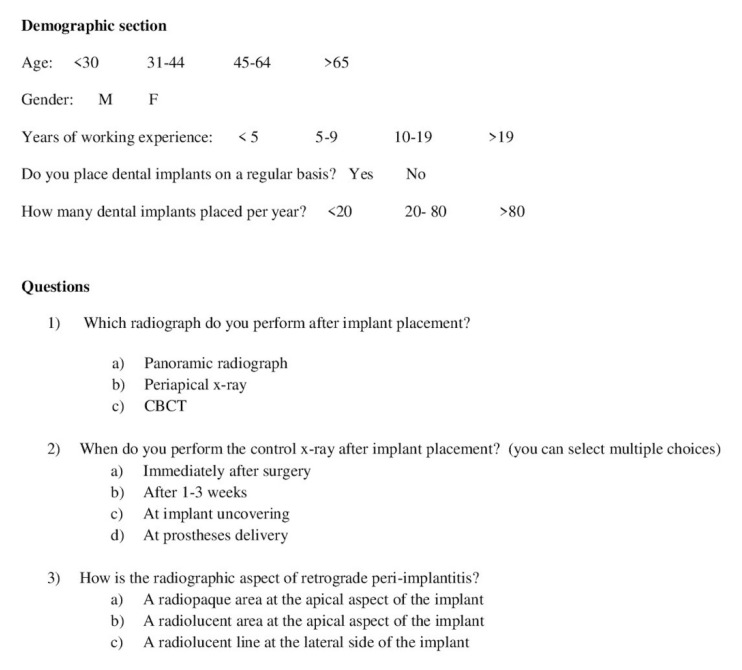
Questionnaire. This file contains the English version of the survey sent to participants.

An anonymous questionnaire regarding radiographic imaging in implant dentistry was sent via email to dentists randomly selected from the official register of implantology and oral surgery Italian scientific societies (Figure 2).

The only requirements considered for inviting potential participants to take the survey were: inclusion in the list of these organizations, and an active email address. In order to be enrolled in this survey, dentists had to place dental implants on a regular basis. The questionnaire was sent to a randomly selected sample of 937 dentists.

### 4.3. Survey

These invitation emails contained an individual link to a web-based questionnaire, which could only be answered once. Reminder emails were sent once a week for the length of the study. The questionnaire was anonymous and no personal data of participants were collected. The survey was divided into two sections, and by agreeing to answer it, participants signed the informed consent form (Figure 2). The first section was about demographic information (age, gender, years of working experience, practice as implantologist, number of dental implants placed per year). The second section included three questions regarding the preferred choice for X-ray after implant placement, the timing of the post-operative assessment, and the knowledge of the radiographic representation of retrograde peri-implantitis. All questions were multiple answer and close-ended, but in one case (question #3), more than one answer were allowed. 

### 4.4. Statistical Analysis

Data were evaluated using standard statistical analysis software (version 20.0, Statistical Package for the Social Sciences, IBM Corporation, Armonk, NY, USA). A database was created using Excel (Microsoft, Redmond, WA, USA). Descriptive statistics of demographic characteristics of participants were provided.

Categorical variables were summarized as frequency and percentage. Chi-squared test and the Fisher exact test were computed to measure bivariate association between personal characteristics (age, years of experience and number of dental implants/year) of participants and correct answers. The cut-off for statistical significance was *p* ≤ 0.05. 

## 5. Conclusions

To the best of the authors’ knowledge, this is the first study to report data on preferences and the attitude of implant practitioners in radiographic imaging after implant placement. Therefore, there are no studies to which our findings can be directly compared. The appropriateness of radiographic prescription habits among dentists is extremely important: radiography is considered the most used diagnostic tool in dentistry, accounting for almost a quarter of total X-rays in the medical field worldwide. The proper and rapid diagnosis of peri-implant diseases and implant post-operative complications is of paramount importance for long-term implant survival and success. Furthermore, establishing an appropriate radiographic baseline for implant evaluation is beneficial also from a medico-legal point of view. The majority of implantologists (84.3%) perform a periapical X-ray as routine control radiograph and chose on the survey the correct radiographic representation of RPI (74.3%), without statistically significant differences (*p* > 0.05) for sex, age, years of working practice or number of implants placed per year. Just 47.7% of dentists perform a control X-ray at the day of prostheses delivery, as recommended by the 2017 World Workshop to establish a proper baseline for radiographic assessment, while the majority of the sample interviewed (52.3%) did not select this answer. Based on the analysis of demographic characteristics, a statistically significant difference (*p* < 0.05) was detectable for the number of implants placed per year, with dentists placing more than 80 implants selecting the correct choice, and no significant differences (*p* > 0.05) for sex, age and years of working experience. 

Future research should be orientated in highlighting the importance of the appropriate timing of control X-ray, with the establishment of proper guidelines.

## Figures and Tables

**Table 1:** Sample demographics.

Variable	N	%
Gender	-	-
Male	386	88.9
Female	48	11.1
Age range	-	-
< 30 years	78	17.9
30–44 years	114	26.3
45–64 years	218	50.3
> 65 years	24	5.5
Years of working experience	-	-
< 5 years	74	17.1
5–9 years	60	13.8
10–19 years	106	24.5
> 19 years	194	44.6
Number of implants/year	-	-
< 20	114	26.2
20–80	214	49.3
> 80	106	24.5

**Table 2:** Detailed results of question #1, divided for sex, age, years of experience and number of dental implants placed per year.

Variable	PAN	Periapical	CBCT	*p* Value			Overall *p* Value
Gender							
Male	36	322	28	0.701	0.158	< 0.0001 *	0.524
Female	2	44	2	0.194	0.469	0.324	
Age range							
< 30 years	2	70	6	0.039 *	0.443	0.945	0.265
30–44 years	10	100	4	0.816	0.060	0.118	
45–64 years	26	174	18	0.154	0.309	0.611	
> 65 years	0	22	2	< 0.0001 *	0.823	0.827	
Years of working experience							
< 5 years	2	66	6	0.048 *	0.535	0.976	0.527
5–9 years	8	50	2	0.515	0.401	0.198	
10–19 years	6	92	8	0.222	0.745	0.957	
> 19 years	22	158	14	0.332	0.215	0.918	
Number of implants/year							
< 20	6	100	8	0.157	0.543	0.813	0.687
20–80	18	180	14	0.694	0.946	0.647	
> 80	14	84	8	0.305	0.176	0.948	

PAN: Panoramic X-ray; Periapical: periapical X-ray; CBCT: Cone-Beam Computed Tomography; * statistically significant (*p* < 0.05).

**Table 3:** Detailed results of question #2, divided for sex, age, years of experience and number of dental implants placed per year.

Variable	Other	Correct	*p* Value		Overall *p* Value
Gender					
Male	200	186	0.996	0.702	0.545
Female	28	20	0.641	0.413	
Age range					
< 30 years	40	36	0.891	0.826	0.316
30–44 years	52	62	0.182	0.305	
45–64 years	117	103	0.897	0.687	
> 65 years	18	6	0.183	0.050 *	
Years of working experience					
< 5 years	40	32	0.809	0.544	0.484
5–9 years	24	36	0.098	0.208	
10–19 years	54	52	0.697	0.942	
> 19 years	109	87	0.451	0.304	
Number of implants/year					
< 20	64	48	0.521	0.340	0.044 *
20–80	124	92	0.191	0.114	
> 80	40	66	0.008 *	0.020 *	

Other: answers not including “at prostheses delivery”, Correct: answers including “at prostheses delivery”; * statistically significant (*p* < 0.05).

**Table 4:** Detailed results of question #3 divided for sex, age, years of experience and number of dental implants placed per year.

Variable	Radiopaque Apical	Radiolucent Apical	Radiolucent Lateral	*p* Value			Overall *p* Value
Gender							
Male	34	292	60	0.747	0.498	0.067	0.248
Female	2	32	14	0.214	0.251	0.187	
Age range							
< 30 years	5	62	11	0.267	0.355	0.328	0.310
30–44 years	7	86	19	0.222	0.111	0.691	
45–64 years	27	154	39	0.098	0.160	0.900	
> 65 years	0	16	8	< 0.0001 *	0.333	0.266	
Years of working experience							
< 5 years	7	62	5	0.773	0.107	0.011 *	0.081
5–9 years	0	46	10	< 0.0001 *	0.070	0.890	
10–19 years	11	72	25	0.948	0.163	0.327	
> 19 years	21	138	37	0.506	0.262	0.789	
Number of implants/year							
< 20	5	88	21	0.051	0.491	0.943	0.107
20–80	25	141	45	0.181	0.016 *	0.214	
> 80	9	89	11	0.599	0.124	0.039 *	

* statistically significant (*p* < 0.05).
